# A Review on the use of Synthetic and Recombinant Antigens for the Immunodiagnosis of Tegumentary Leishmaniasis

**DOI:** 10.2174/0109298673298705240311114203

**Published:** 2024-03-19

**Authors:** Kamila Alves Silva, Anna Júlia Ribeiro, Isadora Braga Gandra, Carlos Ananias Aparecido Resende, Lucas da Silva Lopes, Carolina Alves Petit Couto, Verônica de Araujo Freire, Isabelle Caroline Santos Barcelos, Sabrina Paula Pereira, Sandra Rodrigues Xavier, Mariana Campos da Paz, Rodolfo Cordeiro Giunchetti, Miguel Angel Chávez-Fumagalli, Ana Alice Maia Gonçalves, Eduardo Antonio Ferraz Coelho, Alexsandro Sobreira Galdino

**Affiliations:** 1 Laboratório de Biotecnologia de Microrganismos, Departamento de Bioquímica, Universidade Federal de Sao Joao Del-Rei (UFSJ), Campus Centro Oeste, Divinópolis, 35501-296, Minas Gerais, Brasil;; 2 Laboratório de Bioativos e Nanobiotecnologia, Universidade Federal de São João Del-Rei, Divinópolis, 35501-296, Minas Gerais, Brasil;; 3 Laboratório de Biologia das Interações Celulares, Departamento de Morfologia, Instituto de Ciências Biológicas, Universidade Federal de Minas Gerais, Belo Horizonte, 31270-901, Minas Gerais, Brasil;; 4 Computational Biology and Chemistry Research Group, Vicerrectorado de Investigación, Universidad Católica de Santa María, Arequipa 04000, Peru;; 5 Programa de Pós-Graduação em Ciências da Saúde: Infectologia e Medicina Tropical, Faculdade de Medicina, Universidade Federal de Minas Gerais, Belo Horizonte, 30130-100, Minas Gerais, Brasil

**Keywords:** Tegumentary leishmaniasis, diagnosis, recombinant protein, recombinant multiepitope protein, synthetic peptide, etiologic agents

## Abstract

Improving the diagnostic technology used to detect tegumentary leishmaniasis (TL) is essential in view of it being a widespread, often neglected tropical disease, with cases reported from the Southern United States to Northern Argentina. Recombinant proteins, recombinant multiepitope proteins, and synthetic peptides have been extensively researched and used in disease diagnosis. One of the benefits of applying these antigens is a measurable increase in sensitivity and specificity, which improves test accuracy. The present review aims to describe the use of these antigens and their diagnostic effectiveness. With that in mind, a bibliographic survey was conducted on the PudMed platform using the search terms “tegumentary leishmaniasis” AND “diagno”, revealing that recombinant proteins have been described and evaluated for their value in TL diagnosis since the 1990s. However, there was a spike in the number of publications using all of the antigens between 2013 and 2022, confirming an expansion in research efforts to improve diagnosis. Moreover, all of the studies involving different antigens had promising results, including improved sensitivity and specificity. These data recognize the importance of doing research with new technologies focused on developing quick, more effective diagnostic kits as early diagnosis facilitates treatment.

## 1 INTRODUCTION

Tegumentary leishmaniasis (TL) is a neglected tropical disease, also known in the Americas as American tegumentary leishmaniasis (ATL), and one of the world’s foremost infectious tropical diseases due to the extent of illness it causes in patients [1, 2]. It is considered an emerging disease and an alarming upsurge in its incidence has been reported [3, 4]. Currently, the disease is endemic in 92 countries, with an estimated occurrence of 0.6 to 1.0 million new cases per year [5]. Brazil is among the nine endemic countries with the highest number of TL cases, averaging 21,000 cases annually [5]. The disease is caused by etiologic agents of the *Viannia* and *Leishmania* subgenera, with the main species in the Old World being *Leishmania major*, *L. tropica,* and *L. aethiopica*, and in the Americas, *L. mexicana*, *L. amazonensis*, *L. venezuelensis*, *L. braziliensis*, *L. panamensis,* and *L. guyanensis* [6, 7]. Parasite transmission occurs through the blood meal of female sandflies of the genera *Phlebotomos* and *Luztomyia* in the Old World and the Americas, respectively [8-12]. TL presents clinical symptoms ranging from single or multiple ulcerative cutaneous lesions (cutaneous leishmaniasis - CL), diffuse lesions (diffuse leishmaniasis - DL), and mucosal lesions (mucosal leishmaniasis - ML), the last two being typical in the Americas [13, 14]. It is related to physical deformities and psychological changes that affect the infected individual's health [15, 16]. Additionally, societal stigma and prejudice are attached to the disease, compromising the individual's quality of life and emotional well-being [17]. The variety of clinical symptoms can hinder arriving at an accurate diagnosis, a crucial step for treatment and infection control [10]. In spite of recent advances, diagnosing the disease continues to be based on the triad of epidemiological antecedents, clinical signs, and laboratory tests, including direct and histopathological examination of a skin biopsy and molecular detection of *Leishmania* DNA [18, 19]. Although specificity values are high, sensitivity values have been variable [20, 21]. In addition, molecular techniques are complex, expensive, lack a differentiated protocol for routine use, and are usually restricted to reference and research centers [3]. Serological tests, such as ELISA and rapid tests, offer some benefits over others as they are less invasive, cheaper, easier to perform, and can be applied in the field [22-24]. In this sense, serological diagnosis is an important tool for controlling and preventing infectious diseases. Despite their advantages, the sensitivity and specificity values of these assays may vary, depending especially on the type of antigen [25]. In this context, the development of recombinant antigens, such as recombinant protein (RP), recombinant multiepitope protein (RMP), and synthetic peptides, has proven to be an important alternative for disease diagnosis as they present better sensitivity and specificity values [26, 27]. Furthermore, these antigens have the advantage of working without the need for the strict biosafety requirements needed when handling microorganisms, and are better suited for assay standardization [28]. In fact, some studies demonstrate the high performance of these antigens when applied to human serodiagnosis [26, 29-34] and canine leishmaniasis [35-37]. The aim of this review is to discuss the use of RPs, RMPs, and synthetic peptides for TL diagnosis using the ELISA and rapid tests.

## 2 THE ADVANTAGES OF RECOMBINANT ANTIGENS AND SYNTHETIC PEPTIDES IN SEROLOGICAL DIAGNOSIS

The search for new technologies, including more sensitive and specific antigens, is a necessity for the diagnostic industry as there is no gold standard serological test for TL [38-41]. In this sense, RP, RMPs and synthetic peptides are currently being the focus of many researchers. Fig. (**1**) represents the flowchart of the selection of these antigens. The RP market is on the Rise, with a projected annual growth of around 12% from 2022 to 2030, and estimated to reach $5.09 billion by 2030, [42]. The diagnosis market represents a significant percentage among the segments that use RPs. These antigens are important owing to their diverse applications in chemistry, pharmaceuticals, cosmetics production, human and animal health, agriculture, food industries, and waste treatment [43]. RPs are native proteins produced through genetic engineering techniques, allowing expression in heterologous host systems, such as bacteria or mammalian cells [25]. They can be obtained in large quantities with a high degree of purity [44]. Among their many uses, RP have been described as a powerful tool to diagnose TL and other infectious diseases, such as Chagas disease [45], visceral leishmaniasis [46], tuberculosis [47], toxoplasmosis [48], scrub typhus [49], and leptospirosis [50].

The RMPs that fall under the RP category are constructs that contain multiple epitopes in a single molecule [29]. The construction of these molecules, containing high-density epitopes, is an alternative for disease diagnosis as they have a greater capacity to expose a higher number of antigenic epitopes, resulting in greater sensitivity and specificity [26, 29, 37]. These epitopes are specific sequences that can be recognized by the immune system and used as antigens in immunological assays, allowing the simultaneous detection of different subsets of antibody classes associated with diseases [37]. Furthermore, RMPs offer other advantages aimed at improving diagnostic efficiency, such as low production cost, and easy handling in serological tests [30, 35]. These RMP antigens have already been used to diagnose various diseases, such as toxoplasmosis [51], tuberculosis [52], and hepatitis C [53] with satisfactory results.

The synthetic peptide market in diagnostic assays is expected to grow by 9.6% from 2022 to 2027, with forecasts to reach a value of USD 11.4 billion [54]. Peptides are short amino acid sequences that mimic the specific antigenic regions of native proteins [55]. These molecules can be obtained through chemical synthesis and enzymatic hydrolysis [56], and are used in immunoassays to detect the presence of specific anti-bodies in samples, allowing a quick and accurate diagnosis of different diseases [55-57]. The use of peptides confers advantages, such as low-cost production, as only a single peptide needs to be synthesized in a simplified chemical production process with more controlled conditions, high reproducibility, ease of storage, stability, and safety [55, 58]. In addition, when compared to the use of RPs, studies have shown that synthetic peptides elevate the sensitivity and/or specificity of immunoassays for the serodiagnosis of diseases, such as leishmaniasis [38, 59, 60].

## 3 THE USE OF RECOMBINANT ANTIGENS AND SYNTHETIC PEPTIDES FOR TL SEROLOGICAL DIAGNOSIS USING THE ELISA TECHNIQUE

The ELISA technique is one of the principle tools used in the field of immunology and laboratory diagnosis because of its specificity and sensitivity in detecting biomolecules [61]. ELISA is widely used in many areas, including medical diagnoses, biomedical research, and quality control in the pharmaceutical and food industries, detecting a number of diseases, helping spot the presence of allergens in food, and monitoring biomarkers in clinical studies [62]. It also detects and quantifies specific substances in complex biological samples, such as antigens or antibodies [63]. In the diagnostic field, this assay involves the use of a microtiter plate in which specific antigens or antibodies are fixed on the surface of the wells [62]. These ligands are chosen according to the substance of interest to be detected with the aim of showing antigen-antibody reactions [62]. ELISA has been broadly applied in TL diagnostic research with promising results.

Currently, there are fourteen studies using RPs in the literature, three using RMPs. and seven using epitopes. The results for each one are summarized below.

### 3.1 Recombinant Proteins-based ELISA Assays

The study conducted by Montoya *et al.* (1997) was the first one to use RPs for TL diagnosis. In their word, two RPs were selected, called T26-U2 and T26-U4, which were expressed using Escherichia cells. To evaluate protein's reactivity, a serological panel composed of 250 CL and 18 ML serum samples was employed. In addition, human serum samples that may present a cross-reaction, such as Chagas disease, malaria, bartonellosis, tuberculosis, paracoccidiosis, and sporotrichosis, were used. After performing an ELISA assay to assess protein reactivity, T26-U2 results showed sensitivity and specificity values of 58% and 87%, respectively. In relation to T26-U4 results, 76% sensitivity and 97% specificity were observed. Moreover, T26-U2 and T26-U4 RP were combined in the assay with results showing 87% for both sensitivity and specificity [64]. Rey-Ladino *et al.* (1997) conducted a study to test the diagnostic efficiency of the RP called rLHSP60. The protein’s reactivity with CL serum samples was analyzed, in which all of the recognized rLHSP60. However, serum samples from individuals with visceral leishmaniasis and Chagas disease also recognized the RP. However, sensitivity and specificity values were not shown [65]. Celeste *et al.* (2004) studied the RPs called Hsp83 and Hsp70. The serological panel was composed of 12 CL and 14 ML serum samples from infected individuals, along with 10 serum samples from healthy individuals used as a negative control. Serum from individuals with Chagas disease were used to assess cross-reactivity. Hsp83 showed the best performance as the antigen was recognized by all ML-positive samples and by a large part of the CL samples, while no cross-reactions were observed [66]. Souza *et al.* (2013) conducted a study to evaluate the diagnostic capacity of different RPs, called rHSP70, rH2A, rH2B, rH3, rH4, and rKMP11, using *E. coli* cells for protein expression. The serological panel consisted of 49 CL and 53 ML serum samples, in addition to 39 samples from individuals from endemic and 49 from non-endemic areas used as a negative control. Moreover, serum samples from diseases that could cause cross-reactivity, such Chagas disease, systemic lupus erythematosus, leprosy, and tuberculosis, were used. After performing an ELISA assay, ML serum samples showed greater reactivity to most RPs as compared to CL serum samples, with rHSP70 and rH2A showing the best performance. HSP70 sensitivity values were determined as 83%, 73.5%, and 65% when considering ML, CL, and ML+ CL samples, respectively. Moreover, HSP70 specificity values were calculated at 81.8%, 72.7%, and 92% for ML, CL, and ML+CL samples, respectively. Similarly, rH2A sensitivity and specificity values ranged from 60 to 71.7% and 71.5 to 72.7%, respectively, when considering the different samples [67]. Menezes-Sousa *et al.* (2014) worked with an RP called rHSP83.1, expressed in *E. coli* BL21 (DE3) Arctic cells. In their study, 65 samples from CL or ML were used to assess protein reactivity. In addition, serum samples from individuals with Chagas disease were used to verify possible cross-reactivity. When considering CL serum samples, the ELISA assay results showed a sensitivity value of 95.55%. Regarding ML samples, a 90.00% sensitivity value was determined. Moreover, a rHSP83.1 specificity value of 93.85% was calculated [68]. Subsequently, Menezes-Sousa *et al.* (2015) worked with two RPs, rLbMAPK3 and rLbMAPK4, also expressed in *E. coli* BL21 (DE3) Arctic cells. In their study, 45 CL and 20 ML serum samples were used to evaluate protein reactivity. Again, serum samples from individuals with Chagas disease were used to check for possible cross-reactivity. When considering rLbMAPK3 results, 83.08% sensitivity and 71.43% specificity were observed, considering both CL and ML serum samples. Regarding the rLbMAPK4 diagnostic performance, 75.38% sensitivity and 97.14% specificity were determined [69]. Celeste *et al.* (2014) tested the RP rHsp83, which was expressed using *E*. *coli* M15 cells. To evaluate its reactivity, 12 CL and 14 ML serum samples were used, as well as 30 serum samples from healthy individuals for a negative control. Moreover, serum samples from diseases that could present cross-reactions, such as Chagas disease, blastomycosis, histoplasmosis, aspergillosis, chromomycosis, toxoplasmosis, cytomegalovirus, malaria, and tuberculosis, were used to test for cross-reactivity. The results from the rHsp83-based ELISA showed a 100% sensitivity value for both CL and ML samples and a 97.47% specificity value [70]. Coelho *et al.* (2015) conducted a study to verify the diagnostic capacity of the RPs, rCcOx and rHRF, which were expressed in *E. coli* cells. To evaluate their performance, serological tests were conducted using 12 CL and 12 ML serum samples from infected individuals. Additionally, 20 serum samples from uninfected individuals were used as a negative control, as well as serum from Chagas disease infected individuals to assess possible cross-reactions. Both rCcOx and rHRF showed a good serological performance and demonstrated 100% sensitivity and specificity [71]. Duarte *et al.* (2015) evaluated the diagnostic capacity of tryparedoxin peroxidase, eukaryotic initiation factor 5α, enolase, β-Tubulin, and hypothetical RPs, which were expressed in *E. coli* BL21 (DE3) cells. A serological panel containing 20 ML and 23 CL serum samples was used to evaluate their reactivity. Furthermore, 30 samples from uninfected individuals were used as a negative control, in addition to the use of positive serum samples from diseases that could cause cross-reactions, such as Chagas disease. After conducting an ELISA assay, tryparedoxin peroxidase showed 100% sensitivity and specificity values. Eukaryotic initiation factor 5α showed sensitivity and specificity values of 100.0% and 92.5%, respectively. Regarding recombinant enolase results, 100.0% sensitivity and 85.0% specificity were observed. β- Tubulin showed a value of 100.0% for sensitivity and 82.5% for specificity. Lastly, the recombinant hypothetical protein showed 95.4% sensitivity and 85.0% specificity [72]. Lima *et al.* (2017) developed a study to evaluate the rLbHyM diagnostic capacity. *E. coli* BL21 (DE3) Arctic cells were used for heterologous protein expression. To analyze the diagnostic performance, 25 serum from healthy individuals residing in endemic areas and 25 serum from healthy individuals from non-endemic areas were used as a negative control. Regarding positive serum samples, 20 CL and 25 ML individual serum samples were used. In addition, Chagas disease positive serum samples were used to verify the possibility of cross-reaction between species. rlbHyM-based ELISA results showed sensitivity and specificity of 100% and 98.0%, respectively [73]. Carvalho *et al.* (2017) conducted a study with a RP called rLiHypA, with this protein having been obtained after heterologous expression in *E*. *coli* cells. rLiHypA-diagnostic capability was evaluated using 57 ML and 27 CL serum samples. As a negative control, 40 serum samples from healthy individuals residing in endemic areas were used. In addition, serum from individuals with Chagas disease were used to assess possible cross-reactivity. Sensitivity and specificity values of 100% and 98.2%, respectively, were determined after performing an ELISA assay [74]. In the same year, Sato *et al.* (2017) worked with two RPs, rLb6H and rLb8E, which were expressed in *E. coli* BL21 cells. A total of 219 positive TL and 68 healthy individual serum samples were used to test their serological performance. Moreover, samples from patients with diseases that could present cross-reactions, such as Chagas disease, histoplasmosis, malaria, paracoccidioidomycosis, toxoplasmosis, and tuberculosis, were also tested. rLb6H showed the best results in an ELISA assay, with sensitivity and specificity values of 100.0% and 98.5%, respectively [75]. Lima *et al.* (2018) produced four RPs, rLiHyM, rEnolase, rEIF5a, and rBeta-tubulin, using *E*. *coli* BL21 cells. A serological panel consisting of 15 CL and 25 ML serum samples was used for reactivity analyses, as well as 30 serum samples from healthy individuals from non-endemic areas and 45 samples from healthy individuals from endemic areas used as negative controls. Moreover, serum samples from individuals with Chagas disease, paracoccidioidomycosis, leprosy, and aspergillosis were also used to assess possible cross-reactions. All the tested RPs showed the same sensitivity and specificity values of 100% and 97.78%, respectively [76]. Salles *et al.* (2018) evaluated the diagnostic capacity of a small myristoylated recombinant protein-3 (SMP-3), which was expressed in *E*. *coli* BL21 (DE3). The authors used a serological panel of 25 ML and 15 CL serum samples to assess protein reactivity. Moreover, 35 serum samples from uninfected individuals were used as a negative control, in addition to the use of positive serum from other diseases that could cause cross-reactions, such as Chagas disease, paracoccidioidomycosis, leprosy, and aspergillosis. The authors observed values of 100% sensitivity and 99% specificity after performing an ELISA assay [60]. Ribeiro *et al.* (2018) conducted a study to evaluate the diagnostic capacity of the RP called rLiHyE, using *E. coli* cells for protein expression. The serological panel consisted of 15 CL and 15 ML serum samples, in addition to 20 serum samples from healthy individuals from endemic areas and 20 serum samples from healthy individuals from non-endemic areas, used as negative controls. In addition, samples from diseases that could cause cross-reactivity were used, such as Chagas disease, paracoccidioidomycosis, leprosy, and aspergillosis. rLiHyE-based ELISA sensitivity and specificity values were 100.0% and 98.9%, respectively [77]. Medeiros *et al.* (2022) evaluated the diagnostic potential of the RP triparedoxin peroxidase (TryP). This protein was expressed in *E*. *coli* BL21 (DE3) Arctic cells and CL or ML serum samples from 70 patients were used for the ELISA serological assays. In addition, 70 serum samples were used as a negative control and for cross-reaction. Results showed 88.57% sensitivity and 90% specificity in the rTryP ELISA assay [78].

### 3.2 Recombinant Multiepitope Protein-based ELISA Assays

Despite not being such a new technique, the use of RMP for TL diagnosis remains poorly explored. Garcia *et al.* (2021) published the first study using an RMP for TL diagnosis. After selecting epitopes through bioinformatics analyses, a RMP based on linear B cell epitopes was constructed and called rChip. After obtaining rChip using *E*. *coli* cells, serological assays were performed using 35 CL and 35 ML serum samples, in addition to 35 serum samples from healthy individuals used as a negative control. Serum samples from individuals with Chagas disease were also used to assess possible cross-reactions. Sensitivity and specificity values of 100% were obtained, showing a much better performance when compared to ELISA based on soluble antigens and the IFA commercial kit [41].

Next, Galvani *et al.* (2021) worked with a RMP identified as ChimLeish. After selecting epitopes using bioinformatics analyses, ChimLeish was expressed in *E*. *coli* cells. The serological panel consisted of 25 CL and 30 ML serum samples, in addition to 25 serum from healthy individuals residing in endemic areas used as a negative control. Serum samples from other diseases, such as Chagas disease, leprosy, aspergillosis, histoplasmosis, and HIV, were tested for possible cross-reactions. The ChimLeish-based ELISA assay obtained 100% sensitivity and specificity values [79].

Vale *et al.* (2022) developed a RMP, ChimB, after selecting epitopes through bioinformatics analyses, which was then expressed in *E*. *coli* cells. For serological assays, 25 CL and 25 ML samples were used, and 25 serum from healthy individuals residing in endemic areas were used as a negative control. Serum samples were also tested from patients with Chagas disease, leprosy, aspergillosis, paracoccidioidomycosis, histoplasmosis, and HIV to access cross-reactions. The serological assay results showed 100% sensitivity and specificity [25].

### 3.3 Peptide-based ELISA

The study published by Menezes-Sousa *et al.* (2014) was the first one to use synthetic peptides for TL diagnosis. The authors used bioinformatics analyses to select the peptides to identify linear B cell epitopes. The selected peptides, identified as peptide-1, peptide-2, and peptide-3, were obtained through chemical synthesis. The serological panel was composed of 65 CL or ML serum samples, in addition to serum samples from individuals with Chagas disease to assess cross-reactivity. Peptide-1, peptid-2, and peptide-3 sensitivity values were 71.11%, 64.44%, and 95.55%, respectively, for CL serum samples. Regarding ML serum samples, sensitivity values were estimated as 55.00%, 50.00%, and 75.00% for peptide-1, peptide-2, and peptide-3, respectively. Specificity values were determined as 94.29%, 90.00% and 91.43% for peptide-1, peptide-2, and peptide-3, respectively, with peptide-3 showing the best performance. However, its performance was less impressive when compared to that of the recombinant proteins [68].

Next, Menezes-Sousa *et al.* (2015) used bioinformatics analyses to identify linear B cells epitopes. The selected peptides, peptide-1 and peptide-2, were obtained through chemical synthesis. The serological panel for the ELISA assay was composed of 65 CL or ML serum samples, in addition to 20 serum samples from patients with Chagas disease to evaluate cross-reactivity. The results showed that peptide-1 and peptide-2 had the same diagnostic performance, with sensitivity and specificity values of 98.46% and 95.71%, respectively. Moreover, the synthetic peptides performed better than the RPs [69].

Costa *et al.* (2016) used a phage display technique to select new epitopes for TL diagnosis. Initially, six phage clones were selected, identified as A10, B7, B10, C11, C12, and H7. To further evaluate their reactivity, 20 CL and 30 ML serum samples were used. In addition, 20 serum samples from healthy individuals living in endemic regions and 30 serum samples from healthy individuals living in a non-endemic area were used as a negative control, plus serum samples from individuals infected with Chagas disease and visceral leishmaniasis to assess cross-reactions. The phage-based ELISA results showed that A10, C12, and H7 had the best diagnostic performance, with sensitivity and specificity values of 100% [80].

Salles *et al.* (2018) used bioinformatics analyses to select a peptide for TL diagnosis. After obtaining the peptide using chemical synthesis, an ELISA assay was performed using 15 CL and 25 ML serum samples, as well as 35 serum samples from healthy individuals living in an endemic area, which were used as a negative control. Serum samples were also used from infected individuals that could present cross-reaction, such as Chagas disease, paracoccidioidomycosis, leprosy, and aspergillosis. Sensitivity and specificity values were determined as 94.5% and 92.5%, respectively. Nevertheless, the diagnostic ability of the synthetic peptide was lower as compared to that of the RP [60].

Galvani *et al.* (2021) conducted a study to test the diagnostic capability of eight peptides, all selected through bioinformatics analyses and identified as Pept 1, Pept 2, Pept 3, Pept 4, Pept 5, Pept 6, Pept 7, and Pept 8. After chemical synthesis to obtain the peptides, serological assays were performed using 25 CL and 30 ML serum samples. In addition, 25 samples from healthy individuals living in endemic areas were used as a negative control. Cross-reactions were assessed by using serum samples from individuals with such diseases as Chagas disease, leprosy, aspergillosis, histoplasmosis, and HIV. Synthetic peptides sensitivity and specificity values ranged from 9.1% to 90.9% and 98.3% to 99.1%, respectively. Among them, Pept 5 showed the best performance, with sensitivity and specificity values of 90.9% and 99.1%, respectively. However, although Pept 5 showed good results, all of the peptides proved to be inferior when compared to the chimera’s diagnostic performance [79].

Similarly, Vale *et al.* (2022) used bioinformatics analyses to select peptides for TL diagnosis, which were obtained through chemical synthesis. To test the seven peptides selected, identified as PepA, PepB, PepC, PepD, PepF, and PepG, 25 CL and 25 ML serum samples were used. Moreover, 35 serum from healthy individuals residing in endemic areas were used as a negative control. Cross-reaction analyses were also tested using samples from individuals with Chagas disease, leprosy, aspergillosis, paracoccidioidomycosis, histoplasmosis, and HIV. Sensitivity values ranged from 28.0 to 57.3%, while specificity values ranged from 16.3 to 83.7%. The synthetic peptide’s performance was inferior when compared with the RMP developed in this same study [25].

Medeiros *et al.* (2022) selected a peptide using bioinformatics analyses obtained through chemical synthesis. For the serological assay, 70 CL or ML serum samples were used, as well as 70 serum samples for a negative control and cross-reaction. After performing an ELISA assay, both sensitivity and specificity had values of 94.29%. In contrast with the above-cited peptide articles, the peptides in this study performed better when compared to those the recombinant proteins [78]. Table **1** summarizes the main points of the above-cited studies.

## 4 DISCUSSION

TL is a neglected disease primarily endemic to developing countries, where the federal government generally has limited health-related resources [1]. The gold standard for TL diagnosis continues to be the parasitological method [81], where the ideal sample for testing will depend on the causative species and the clinical form [82]. However, due to non-isolation or non-visualization of the parasite, clinical diagnosis and epidemiological data can be also necessary [81, 83]. Moreover, since test efficiency depends on the parasite load, diagnostic accuracy using these methods may be affected, especially when applied to infected individuals with a low parasite burden [84].

The need is growing for a quick, efficient diagnostic tool with good sensitivity and specificity, as is the challenge to find an accurate diagnostic method [10]. Several researchers have conducted serological tests with different types of antigens to improve sensitivity and specificity, which, in turn, would also improve diagnostic accuracy [85]. However, a serological TL diagnosis faces obstacles, mainly due to the low levels of antibodies found in infected individuals, generating false-negative results. CL infected individuals may have low antileishmanial serology, which could lead to false-negative results. ML infected individuals could also present low antileishmanial antibody production, although to a lesser extent [67, 71, 86]. Furthermore, antibodies can remain active for months after disease treatment, which may make it difficult to detect both new and past infections [73, 87].

In that regard, RPs, RMPs, and synthetic peptides are being tested as a way to circumvent these problems. So far, RPs are the most frequently tested antigens in the field of TL diagnosis. However, RMP studies, though smaller in number, have produced the best results, with all studies showing 100% sensitivity and specificity values with the different multiepitopes tested. Furthermore, some studies using RPs and peptides have also shown 100% sensitivity and specificity. Regarding peptides-based serological diagnosis, despite being much smaller in number when compared to recombinant antigens, some studies have demonstrated promising results, with sensitivity and specificity above 90%. Therefore, it is plausible to state that the path to better targeting the choice of antigens to be used in new diagnostic tests is ambiguous, since all categories of referenced recombinant antigens have shown promising results. Moreover, it is not possible to infer which type of antigen is more promising since each study used different methods and different serological panels, which strongly influenced the results.

Although the studies mentioned above demonstrate great potential for better serological diagnosis of the disease, there are factors that must be considered, as they influence the results of diagnostic studies. It is known that species of the genus *Leishmania* present great diversity, especially due to the wide geographic distribution of the same species [88]. This broad parasite genetic diversity can negatively interfere with the diagnostic performance, impacting in the tests’ sensitivity. In addition, the genetic diversity of each tested population can also interfere in diagnostic’s results, since lifestyle, nutritional and immunological status, as well as previous disease history, are important factors that can impact directly in the diagnosis. In this sense, it is essential and urgent to carry out multicenter studies, aiming to increase the accuracy of serological tests and expand the disease detection capacity, even in different geographic regions. Furthermore, aiming at better planning of studies, as well as in order to obtain a more reliable result, the use of tools for calculating the samples to be tested must be taken into account [89].

Another important point to be highlighted, despite being an important tool for serological diagnosis and already being used for several diseases, greatly assisting in a quick and simple diagnosis, rapid test platforms for TL diagnosis still do not yet exist. This might be due to several factors, such as a lack of financial incentives considering leishmaniasis is seen as a neglected illness [90]. Additionally, the lack of awareness among the affected populations, and the distance to the nearest healthcare facilities may conceal the disease’s true effects, exerting an impact on research funding decisions. Indeed, additional incentives for research and clinical studies are needed to validate new diagnostic tests and their effectiveness. Researchers are currently involved in developing alternative diagnostic methods for different diseases with the aim of improving such tests. A notable example is lateral flow biosensors, which show considerable promise due to their sensitivity, specificity, accessibility, speed, robustness, and independence of equipment. These devices offer an innovative approach to disease detection and monitoring, offering significant advantages over traditional diagnostic methods [91, 92]. Furthermore, this platform assay are designed to provide swift results and can be directly performed in the field without the need to send samples to central laboratories, thus allowing a faster diagnosis [93]. Moreover, researchers have also been continuously improving the ELISA platform as a diagnostic tool [94].

Another point to be considered is the choice of antigen, which is fundamental to developing a successful diagnostic test. Several methods are currently available, assisting in better targeting for antigen selection, such as bioinformatics analyses and phage display. Bioinformatics analyses identify pathogens at a lower cost and in less time since the antigens of pathogens can be identified, without the need to manipulate the microorganism [95, 96]. Furthermore, phage display have been widely used in the discovery and development of new characterizations of protein-protein interactions and in epitope mapping. Their versatility and efficiency make it a powerful tool for the study and practical application of protein interactions, increasing yields and reducing costs [97-99].

It is crucial to improve the effectiveness of TL diagnostic tests, allowing early detection, monitoring its spread, and contributing to the development of more effective preventive and therapeutic measures [100]. According to the World Health Organization [4], there are several initiatives underway to improve the diagnosis of neglected diseases. WHO and its partners are working to strengthen health systems in affected countries by improving access to accurate and timely diagnoses. Some of the highlighted efforts are (*i*) development of accessible diagnostic tests, (*ii*) improvement of surveillance systems, which involves training health professionals to recognize the symptoms of these diseases and report cases to health authorities, and (*iii*) the use of innovative technologies, partnerships, and collaborations, with WHO working in partnership with academic institutions, non-governmental organizations, the private sector, and governments to promote research and development of new diagnostic technologies. These partnerships seek to advance research and pool resources to combat these conditions. It is, therefore, reasonable to anticipate the creation of a more precise diagnostic test for TL in the near future.

## CONCLUSION

This review brings together information on the use of recombinant antigens and synthetic peptides appplied in the serological diagnosis of TL infected individuals. In summary, most studies were efficient and demonstrated good sensitivity and specificity results, showing promise as a means for providing an effective diagnosis. Finally, even with such promising results, there is an ongoing need to search for new antigens to develop a quick and effective test.

## Figures and Tables

**Fig. (1):**
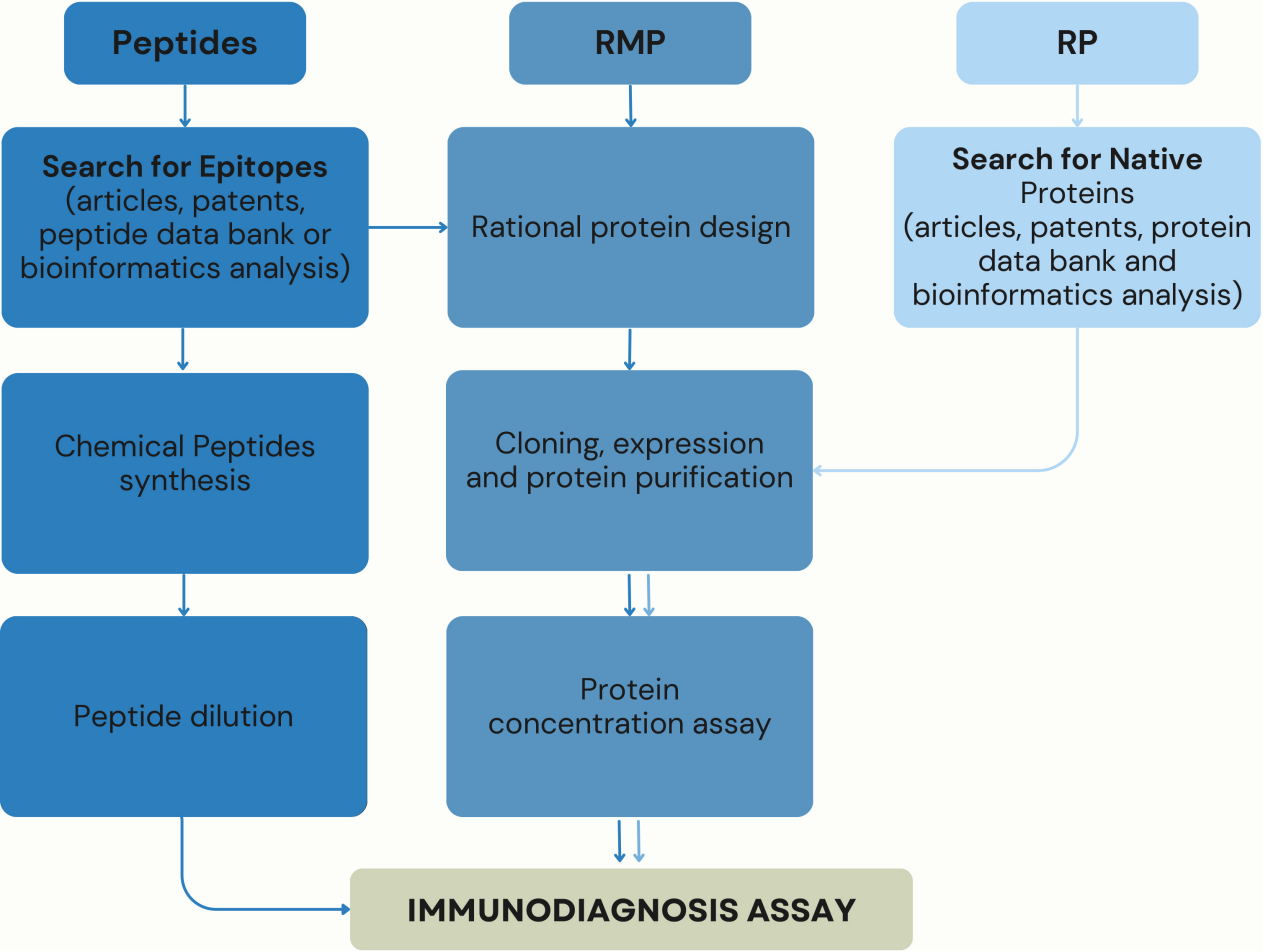
Flowchart of selection and use of peptides, recombinant multiepitope proteins (RMP) and recombinant proteins (RP) for immunodiagnosis. **Abbreviatios: **RMP: recombinant multi-epitope protein; RP: recombinant protein.

**Table 1:** Recombinant antigens and synthetic peptides applied in TL diagnosis using the ELISA method.

**Antigen Type/ Name**	**Heterologous Expression Platform**	**Clinical Manifestations**	**Serological Panel (Positive/ Negative/ Cross-reaction Serum Samples)**	**Results**	**References**
Recombinant protein/ T26-U2 and T26-U4	*E. coli*	CL and ML	145 TL-positive SS Cross-reactive diseases SS: 16 Chagas disease 8 malaria 2 bartonellosis 2 tuberculosis 9 paracoccidiosis 2 sporotrichosis	T26-U2: Sensitivity: 58.0%/ Specificity: 87.0% T26-U4: Sensitivity: 76.0%/ Specificity: 97.0% T26-U2 +T26-U4: Sensitivity: 87.0%/ Specificity: 87.0%	Montoya *et al.*, 1997 [64]
Recombinant protein/ rLHSP60	*E. coli* BL21 (DE3)	CL	TL positive SS (number not informed) 6 TL-negative SS Cross-reactive diseases SS: Chagas disease SS (number not informed)	rLHSP60 was recognized by CL and Chagas disease serum samples	Rey-Ladino *et al.*, 1997 [65]
Recombinant protein/ rHsp 83 and rHsp 70 Peptide/50-mer	*E. coli*	CL and ML	26 TL-positive SS 10 TL-negative SS Cross-reactive diseases SS: 10 Chagas disease	rHsp 83, rHsp 70 50-mer showed cross- reactivity with serum from individuals with Chagas disease	Celeste *et al.*, 2004 [66]
Recombinant protein/ rHSP70, rH2A, rH2B, rH3, rH4, and rKMP11	*E. coli*	CL and ML	102 TL-positive SS 88 TL-negative SS Cross-reactive diseases SS: 30 Chagas disease 10 systemic lupus erythematosus 30 leprosy 22 tuberculosis	rHSP70 CL: Sensitivity: 73.5%/ Specificity: 72.7% rHSP70 ML: Sensitivity: 83.0%/ Specificity: 81.8% rHSP70 ML+ CL: Sensitivity: 65.0%/ Specificity: 92.0% rH2A CL: Sensitivity: 71.7%/ Specificity: 71.5% rH2A ML: Sensitivity: 60.0% / Specificity: 72.7% rH2A ML+ CL: Sensitivity: 65.7%/ Specificity: 71.5%	Souza *et al.*, 2013 [67]
Recombinant protein/ rHSP83.1 Peptides/ Peptides 1, 2, and 3	*E. coli* BL21 (DE) Arctic Express	CL and ML	65 TL-positive SS 50 TL-negative SS Cross-reactive diseases SS: 20 Chagas disease	Rhsp83.1 CL: Sensitivity: 95.55%/ Specificity: 93.85% Rhsp83.1 ML: Sensitivity: 90.0%/ Specificity: 93.85% Peptide 1 CL: Sensitivity: 71.11%/ Specificity: 94.29% Peptide 1 ML: Sensitivity: 55.0%/ Specificity: 94.29% Peptide 2 CL: Sensitivity: 64.44%/ Specificity: 90,0% Peptide 2 ML: Sensitivity: 50.0%/ Specificity: 90.0% Peptide 3 CL: Sensitivity: 95.55%/ Specificity: 91.43% Peptide 3 ML: Sensitivity: 75.0%/ Specificity: 91.43%	Menezes-Souza *et al.*, 2014 [68]
Recombinant protein/ rLbMAPK3 and rLbMAPK4 Peptide/ Peptide-1 and peptide-2	*E. coli* BL21 (DE) Arctic Express	CL and ML	65 TL-positive SS 50 TL-negative SS Cross-reactive diseases SS: 20 Chagas disease	MAPK3 TL: Sensitivity: 83.08%/ Specificity: 71.43% MAPK4 TL: Sensitivity: 75.38%/ Specificity: 97.14% Peptide-1 and Peptide-2 TL: Sensitivity: 98.46%/ Specificity: 95.71%	Menezes-Souza *et al.*, 2015 [69]
Recombinant protein/ rHsp83	*E. coli* (M15)	CL and ML	26 TL-positive SS 30 TL-negative SS Cross-reactive diseases SS: 23 Chagas disease 7 blastomycosis 6 histoplasmosis 5 aspergillosis 7 chromomycosis 14 toxoplasmosis 4 cytomegalovirus 9 malaria 4 tuberculosis	Sensitivity: 100 .0%/ Specificity: 97.47%	Celeste *et al.*, 2014 [70]
Recombinant multiepitope protein/ rCcOx and rHRF	*E. coli* BL21	CL and ML	24 TL-positive SS 20 TL-negative SS Cross-reactive diseases SS: 8 *T. cruzi*	Sensitivity: 100.0%/ Specificity: 100.0%	Coelho *et al.*, 2015 [71]
Recombinant protein/ Tryparedoxin peroxidase, Eukaryotic initiation factor 5α, Enolase, β-Tubulin, and Hypothetical protein	*E. coli* BL21	CL and ML	43 TL-positive SS 30 TL-negative SS Cross-reactive diseases SS: 10 Chagas disease	Tryparedoxin peroxidase: Sensitivity: 100.0%/ Specificity: 100.0% Eukaryotic initiation factor 5α: Sensitivity:100.0%/ Specificity: 92.5% Enolase: Sensitivity:100.0%/ Specificity: 85.0% β-Tubulin: Sensitivity:100.0%/ Specificity: 82.5% Hypothetical protein: Sensitivity:95.4%/ Specificity: 85.0%	Duarte *et al.*, 2015 [72]
Peptide/ A10, B7, B10, C11, C12, and H7	*E. coli*	CL and ML	50 TL-positive SS 50 TL-negative SS Cross-reactive diseases SS: 10 Chagas disease	A10, C12, and H7: Sensitivity:100.0%/ Specificity: 100.0% B7: Sensitivity: 66.0% Specificity: 100.0% B10: Sensitivity: 76.00%/ Specificity: 100.0% C11: Sensitivity: 68.0%/ Specificity: 100.0%	Costa *et al.*, 2016 [80]
Recombinant protein/ rLbHyM	*E. coli*	CL and ML	45 TL-positive SS 50 TL-negative SS Cross-reactive diseases SS: 10 Chagas disease	Sensitivity: 100.0%/ Specificity: 98.0%	Lima *et al.*, 2017 [73]
Recombinant protein/ rLiHypA	*E. coli*	CL and ML	57 TL-positive SS 40 TL-negative SS Cross-reactive diseases SS: 15 Chagas disease	Sensitivity: 100.0%/ Specificity: 98.2%	Carvalho *et al.*, 2017 [74]
Recombinant protein/ rLb6H and rLb8E	*E. coli* BL-21	CL and ML	219 TL-positive SS 68 TL-negative SS Cross-reactive diseases SS: 91 Chagas disease 4 histoplasmosis 14 malaria	rLb6H: Sensitivity: 100.0%/ Specificity: 98.5% rLb8E: Sensitivity: 83.3%/ Specificity: 83.3%	Sato *et al.*, 2017 [75]
-	-	-	22 paracoccidioidomycosis 69 toxoplasmosis 13 tuberculosis	-	-
Recombinant protein/ rLiHyM, rEnolase, rEIF5a, and rBeta-tubulina	*E. coli*	CL and ML	40 TL-positive SS 75 TL-negative SS Cross-reactive diseases SS: 30 Chagas disease 8 paracoccidioidomycosis 20 leprosy 10 aspergillosis	rLiHyM, rEnolase, rEIF5a, and rBeta-tubulina: Sensitivity: 100.0%/ Specificity: 97.78%	Lima *et al.*, 2018 [76]
Recombinant protein/ Rsmp-3 Peptide/ -	*E. coli* BL21 (DE3)	CL and ML	50 TL-positive SS 35 TL-negative SS Cross-reactive diseases SS: 30 Chagas disease 30 paracoccidioidomycosis 15 leprosy 10 aspergillosis	rSMP-3: Sensitivity: 100.0% / Specificity: 99.0% Peptide: Sensitivity: 94.5% / Specificity: 92.5%	Salles *et al.*, 2018 [60]
Recombinant protein/ LiHyE	*E. coli* BL21	CL and ML	30 TL-positive SS 40 TL-negative SS Cross-reactive diseases SS: 20 Chagas disease 10 paracoccidioidomycosis 10 leprosy 10 aspergillosis	Sensitivity: 100.0% / Specificity: 98.9%	Ribeiro *et al.*, 2018 [77]
Recombinant multiepitope protein/ rChiP	*E. coli*	CL and ML	70 TL-positive SS 35 TL-negative SS Cross-reactive diseases SS: 35 Chagas disease	Sensitivity: 100.0%/ Specificity: 100.0%	Garcia *et al.*, 2021 [41]
Recombinant multiepitope protein/ rChimLeish Peptide/ 1,2,3,4, 5,6,7, and 8	*E. coli*	CL and ML	55 TL-positive SS 25 TL-negative SS Cross-reactive diseases SS: 25 Chagas disease 20 leprosy 15 aspergillosis 15 histoplasmosis 15 HIV	rChimLeish: Sensitivity: 100.0%/ Specificity: 100.0% Peptide 1: Sensitivity: 25.5%/ Specificity: 98.3%/ Peptide 2: Sensitivity: 29.1%/ Specificity: 99.1% Peptide 3: Sensitivity: 56.4%/ Specificity: 99.1% Peptide 4: Sensitivity: 47.3%/ Specificity: 99.1% Peptide 5: Sensitivity: 90.9%/ Specificity: 99.1%	Galvani *et al.*, 2021 [79]
-	-	-	-	Peptide 6: Sensitivity: 43.6%/ Specificity: 99.1% Peptide 7: Sensitivity: 9.1%/ Specificity: 99.1% Peptide 8: Sensitivity:23.6%/ Specificity: 99.1%	-
Recombinant protein multiepitope / rChimB Peptide/ PepA, PepB, PepC, PepD, PepE, PepF, and PepG	*E. coli* Arctic Express	CL and ML	75 TL-positive SS 35 TL-negative SS Cross-reactive diseases SS: 25 Chagas disease 25 leprosy 10 aspergillosis 10 paracoccidioidomycosis 10 histoplasmosis 20 HIV	rChimB: Sensitivity: 100.0%/ Specificity: 100.0% PepA: Sensitivity: 40.0%/ Specificity: 45.9% PepB: Sensitivity: 34.7%/ Specificity: 78.5% PepC: Sensitivity: 57.3%/ Specificity: 31.9% PepD: Sensitivity: 44.0%/ Specificity: 78.5% PepE: Sensitivity: 52.0%/ Specificity: 37.8% PepF: Sensitivity: 50.7%/ Specificity: 16.3% PepG: Sensitivity: 28.0%/ Specificity: 83.7%	Vale *et al.*, 2022 [25]
Recombinant protein/ rTryP Peptide/ -	*E. coli*	CL and ML	70 TL-positive SS 70 TL-negative SS Cross-reactive diseases SS: Chagas disease (quantity not informed)	RtryP: Sensitivity: 88.57%/ Specificity: 90.0% Peptide: Sensitivity: 94.29%/ Specificity: 94.29%	Medeiros *et al.*, 2022 [78]

**Abbreviations: **CL: cutaneous leishmaniasis; TL: tegumentary leishmaniasis; ML: mucosal leishmaniasis; SS: serum samples; -: information not provided.

## LIST OF ABBREVIATIONS

ATL: American Tegumentary Leishmaniasis
DL: Diffuse Leishmaniasis
CL: Cutaneous Leishmaniasis
ML: Mucosal Lesions
RMP: Recombinant Multiepitope Protein
RP: Recombinant Protein
SS: Serum Samples
TL: Tegumentary Leishmaniasis
